# Design and Characterization of Backside Termination Structures for Thick Fully-Depleted MAPS

**DOI:** 10.3390/s21113809

**Published:** 2021-05-31

**Authors:** Thomas Corradino, Gian-Franco Dalla Betta, Lorenzo De Cilladi, Coralie Neubüser, Lucio Pancheri

**Affiliations:** 1 Dipartimento di Ingegneria Industriale, Università degli Studi di Trento, 38123 Trento, Italy; gianfranco.dallabetta@unitn.it (G.-F.D.B.); lucio.pancheri@unitn.it (L.P.); 2 Trento Institute for Fundamental Physics and Applications–Istituto Nazionale di Fisica Nucleare (TIFPA-INFN), 38123 Trento, Italy; coralie.neubueser@tifpa.infn.it; 3 Dipartimento di Fisica, Università degli Studi di Torino, 10125 Torino, Italy; lorenzo.decilladi@unito.it; 4 Istituto Nazionale di Fisica Nucleare (INFN), Sezione di Torino, 10125 Torino, Italy

**Keywords:** MAPS, radiation detectors, CMOS, device characterization, TCAD simulation

## Abstract

Fully Depleted Monolithic Active Pixel Sensors (FD-MAPS) represent an appealing alternative to hybrid detectors for radiation imaging applications. We have recently demonstrated the feasibility of FD-MAPS based on a commercial 110 nm CMOS technology, adapted using high-resistivity substrates and backside post-processing. A p/n junction diode, fabricated on the detector backside using low-temperature processing steps after the completion of the front-side Back End of Line (BEOL), is reverse-biased to achieve the full depletion of the substrate and thus fast charge collection by drift. Test diodes including termination structures with different numbers of floating guard rings and different pitches were fabricated together with other Process Control Monitor structures. In this paper, we present the design of the backside diodes, together with results from the electrical characterization of the test devices, aiming to improve understanding of the strengths and limitations of the proposed approach. Characterization results obtained on several wafers demonstrate the effectiveness of the termination rings in increasing the breakdown voltage of the backside diodes and in coping with the variability of the passivation layer characteristics. A breakdown voltage exceeding 400 V in the worst case was demonstrated in devices with 30 guard rings with 6 μm pitch, thus enabling the full depletion of high-resistivity substrates with a thickness larger than or equal to 300 μm. Additionally, we show the first direct comparison for this technology of measured pixel characteristics with 3D TCAD simulations, proving a good agreement in the extracted operating voltages.

## 1. Introduction

Monolithic Active Pixel Sensors (MAPS) are considered promising candidates for the replacement of standard hybrid pixel radiation detectors in medical imaging, high-energy physics and space applications due to their advantages in terms of production costs and low material budget [1,2,3,4]. Embedding on the same substrate the radiation sensing element and the conditioning electronics, MAPS need no bump bonding to connect two different silicon substrates and thus allow the fabrication costs to be reduced [1].

In the last few years several monolithic radiation detectors were developed following different approaches. The MAPS produced for the upgrade of the ALICE Inner Tracking System at CERN represent one of the first examples of monolithic sensors applied to a large high-energy physics experiment [5]. In these detectors, the main charge collection mechanism is diffusion, thus limiting their radiation tolerance and speed. Starting from this technology, a new generation of MAPS-based detectors with charge collection dominated by drift is being developed [6,7]. The fabrication of these sensors relies on a modification of a standard 180 nm CMOS process including a deep n-type implantation which enables fully depleting the epitaxial layer. Operating in full depletion, these devices exploit the drift mechanism to speed up the charge collection and improve their radiation hardness [6,7]. In [8,9] the Silicon On Insulator (SOI) approach is presented as an alternative technology for the fabrication of FD-MAPS, where a buried oxide layer is interposed between a thick active layer with high resistivity and a thin microelectronics grade layer. Although this structure enables the two silicon layers to be optimized independently, these devices are affected by charge accumulation in the buried oxide and need a dedicated solution to mitigate this effect. As a result, SOI MAPS require a more complex production process compared to other monolithic technologies, and their portability to more advanced process nodes is reduced.

High-Voltage CMOS (HV-CMOS) processes provide an alternative solution, fully compatible with foundry standard production flows, for the fabrication of FD-MAPS [10]. Several prototypes of radiation pixel sensors have been presented with this approach, having depletion depths as large as several hundreds of μm and an excellent radiation hardness [11]. The main disadvantages of HV-CMOS FD-MAPS are the large capacitance of the sensing electrode and the lower limit of the pixel size, determined by the applied sensor bias.

Sensors reaching several tens of μm depleted thickness with small pixels and small-capacitance electrodes have also been demonstrated [12,13]. For these sensors, different embodiments have been proposed, with the depletion region starting either from the top side [12] or from the bottom side [13]. In this latter case, although not strictly necessary in thin and medium thickness substrates, up to 50 and 200 μm, respectively [13], termination structures should be integrated for improved reliability in thick sensors [14].

In order to provide a cost-effective solution, which could be easily portable to a more scaled process node, we focused on the development of FD-MAPS fabricated with a standard CMOS production process, with a backside junction obtained with low-temperature processing steps carried out after the completion of the entire front-side process [15]. In the first production run, both active pixel arrays with embedded electronics and passive test structures, designed to extract the device characteristic parameters and to characterize the sensor backside diodes, were included. Simulation and characterization results on active pixel arrays and test structures fabricated with this process were reported in [16]. Since a high voltage bias has to be applied to the backside junction to reach full depletion in thick silicon substrates, dedicated termination structures were designed to avoid an early breakdown of the backside junction. This work covers the design and the characterization of the aforementioned backside structures. The backside process is carried out after the completion of the BEOL layers, when high-quality thermal oxide and high-temperature activation of dopants at the back-side cannot be used. To cope with the low-temperature processing requirement, the dopants implanted in the backside surface are activated by laser annealing and the passivation layers are deposited with low-temperature deposition techniques. These passivation layers feature an equivalent trapped charge in the order of 10^12^ cm^−2^, whose concentration cannot be accurately controlled, and a concentration of surface states typically larger than the one present with thermal oxide. Thus, the design of the backside diode termination structures was carried out taking into account the possible high variability in the characteristics of the backside interface and oxide charge. The validation of the backside structures presented in this work is essential to fully understand the possible issues and the characteristics of the employed production process in view of its future use in the fabrication of thick detectors.

In this study, we present the extensive experimental results obtained on the test structures embedded on the backside of a batch of samples produced with a modified 110 nm commercial CMOS process. The influence of the termination structure geometry on the breakdown voltage of the backside junction was quantified by measuring a group of test diodes specifically designed for this purpose. These results were compared with the breakdown voltages estimated from TCAD simulations to validate the simulation models. Gated diodes were characterized to extract the surface generation velocity, while the capacitance-voltage (C–V) characteristics were recorded on backside MOS capacitors to estimate the oxide charge concentration. Finally, we measured the current–voltage (I–V) characteristics of a pixel array without embedded electronics to extract the voltages associated to the full depletion of the device substrate and to the onset of the punch-through between the frontside and backside p-type implants.

The paper is organized as follows. The simulation domains and the models employed in the TCAD simulations are described in Section 2. Section 3 presents the test procedures adopted in the measurements and the methods used to extract the characteristic parameters. In Section 4, we show and compare the results of the TCAD simulations with the results of the tested devices. Finally, we present our conclusions and describe the future perspectives in Section 5.

## 2. TCAD Simulations

**Figure 1:**
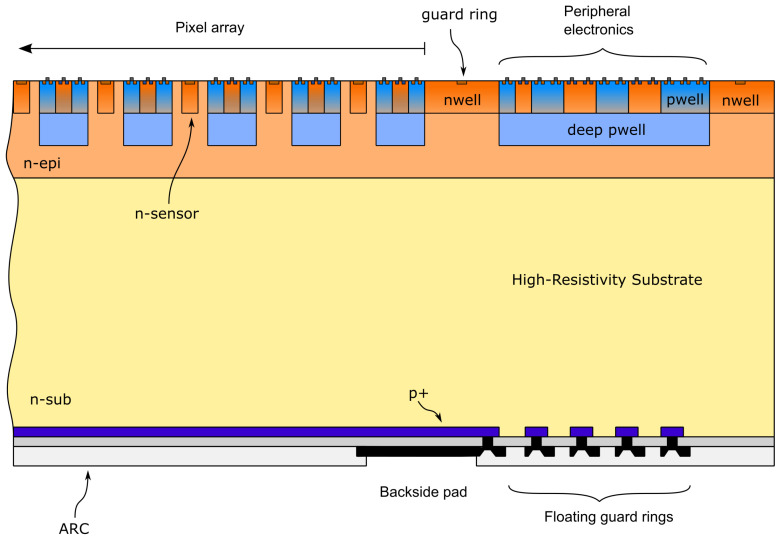
Schematic cross-section of the pixel array with floating backside termination structures.

A schematic cross-section of the proposed sensor including the backside termination structures is shown in Figure 1. The collection electrodes, produced using standard nwell implantations, are located on an n-type epitaxial layer that was grown on top of a high-resistivity (2 k Ω × cm) n-type substrate. The pixel array is surrounded by an n-type guard ring that can be biased to shield the pixels from the current components generated in the surrounding regions. Deep pwells are implanted on the sensor frontside around the collection electrodes and on the array periphery below the nwells and pwells where the in-pixel and peripheral CMOS circuits are hosted. The sensor p–n junction and the floating guard rings, which protect the junction from early edge breakdown, are realized on the wafer backside with $p^{+}$ implantations. A passivation layer is present at the backside surface to protect the floating guard rings, which are equipped with a metal field plate to mitigate the peaks of the electric field at the junctions [17].

A dedicated set of TCAD simulations was carried out to select the most promising geometries for the termination structures in order to prevent breakdown in the operating voltage range. For the considered devices, the lower bound of this range is represented by the voltage needed for the full depletion of the sensor substrate, while the upper bound is related to the voltage at the onset of the punch-through between the frontside pwells/deep pwells, and the backside $p^{+}$ implant.

The design of guard rings for silicon detectors is a known art, covered for example in [18,19]. However, in our case, the optimization had to deal with two additional constraints, arising from the need of processing the backside after the completion of the BEOL at the frontside. The first constraint is the shallow depth of the $p^{+}$/n junction, as a result of the laser annealing used for boron activation at the backside. The second constraint is the density of charges and surface traps that are present at the backside interface, which are much larger than the typical values found in thermal oxide, since the backside passivation layers were deposited using “cold” deposition techniques. These constraints are not typically present in conventional silicon detectors, and thus the design required a thorough optimization.

**Figure 2:**
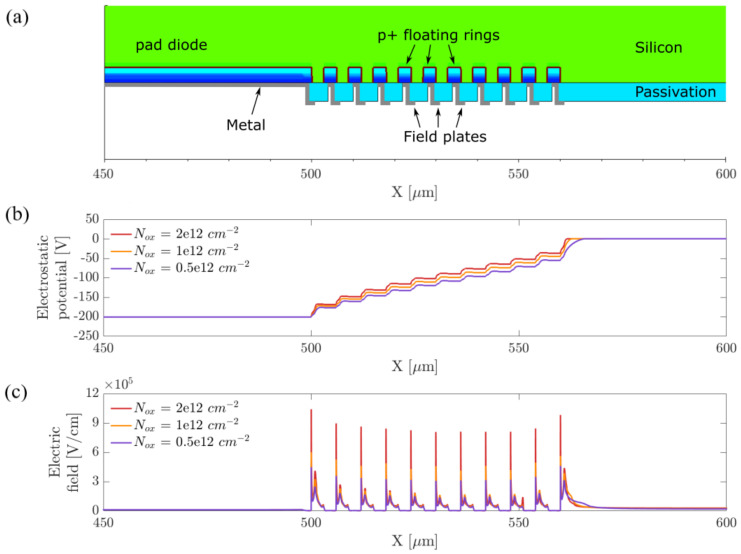
(**a**) Cross section of a guard ring structure in a simplified simulation domain. The green and the blue regions represent the low doped n-type silicon substrate and the $p^{+}$ implants of the backside guard rings and pad diode. The passivation layer is shown in light blue and the gray lines represent the metal field plates. (**b**) Electrostatic potential near the backside surface for three oxide charge concentrations. (**c**) Electric field near the backside surface for three oxide charge concentrations.

The simulations were performed with the Synopsys^®^ Sentaurus TCAD tool. In the first set of simulations we considered a 2D domain representing a simplified cross section of the device and including a variable number of floating rings with different pitches. Figure 2a shows a magnified view of the backside guard ring structure in an example domain with 10 guard rings with a width of 3 μm and a pitch equal to 6 μm. The blue regions represent the $p^{+}$ doped regions of the pad diode and the guard rings. The complete 2D simulation domain, representing a cross section of the produced test structures, is composed of a low doped n-type substrate with a thickness of 300 μm, a total width of 800 μm, and a depth of 1 μm. The $p^{+}$ implant of the pad diode extends over 500 μm, with 300 μm distance between the edge of the pad and the right boundary of the simulation domain.

A thin passivation layer was included on top of the backside surface. The thickness of the insulator was chosen according to the value obtained experimentally from process-control structures. Outward metal field plates were included on top of the guard rings, in order to lower the local electric field peaks and further increase the breakdown voltage of the diode. The surface generation velocity $s_{0}$ at the interface between the silicon substrate and the backside passivation layer was set to 200 cm/s. The value chosen for $s_{0}$ represents an average of the surface generation velocity extracted from process-control structures, as described in the following sections.

We considered different concentrations of charges trapped inside the surface passivation layer $N_{ox}$ from $0.5\times 10^{12}$ to $2\times 10^{12}$ cm^−2^, since the value of this parameter in the deposited layers is subject to large process variations, and the termination structures should be functional within the entire range of variability.

For the estimation of the diode breakdown voltage, we have used the Van Overstraeten and De Man ionization coefficient model [20] based on the Chynoweth law [21]. A negative voltage sweep was applied to the backside electrode until reaching the junction breakdown. The simulations were performed in a quasi stationary regime, where the Drift–Diffusion model and the continuity equations were solved numerically at each step of the voltage sweep.

The two graphs reported in Figure 2b,c represent the electrostatic potential and the electric field along a horizontal cut-line near the backside surface for three different oxide charge concentrations with an applied bias of 200 V. The first plot shows a step-like behavior with an increasing potential difference between the guard rings that are farther from the junction region. Relevant electric field peaks can be observed in the second plot at the p–n junction end and at the guard ring boundaries, and a clear correlation between the amplitude of the electric field peaks and the concentration of oxide charges can be noticed. A double peak is present in correspondence to each guard ring with the first peak located at the boundary of the guard ring junction and the second one at the field plate end.

The breakdown voltages were extracted from the I–V curves, considering a threshold on the diode current equal to $10^{-10}$ A/μm, as a function of the number of guard rings (0, 10, 20, 30), as well as of the guard ring pitch, varying from 5 to 8 μm. In the simulations with variable number of guard rings, the pitch was kept constant and equal to 6 μm, while in the simulations with variable pitch, the number of guard rings was fixed to 10.

**Figure 3:**
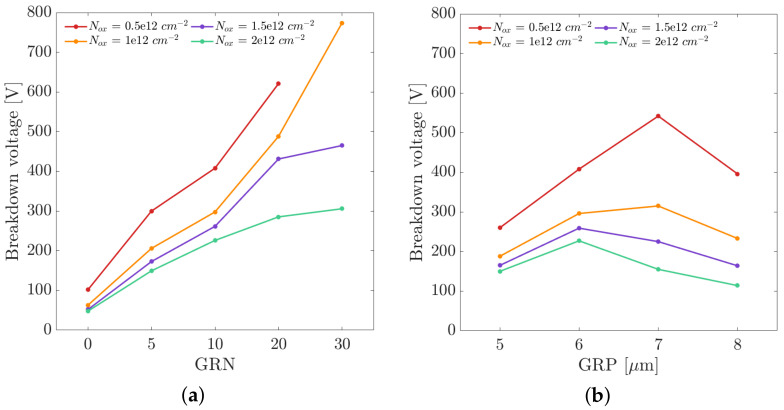
(**a**) Breakdown voltage as a function of the number of guard rings (GRN) for different concentrations of charges in the passivation layer ($N_{ox}$). The results refer to simulations with guard ring pitch equal to 6 μm. (**b**) Breakdown voltage as a function of the guard ring pitch (GRP) for different concentrations of charges in the passivation layer ($N_{ox}$). The results refer to simulations with 10 guard rings.

Figure 3a shows the obtained breakdown voltages as a function of the number of guard rings for four different concentrations of $N_{ox}$. As expected, increasing the number of guard rings leads to a significant increase in the breakdown voltage, and 20 guard rings seem to guarantee a breakdown voltage higher than 250 V for all values of $N_{ox}$ considered. In Figure 3b, where the breakdown voltages are plotted as a function of the guard ring pitch, the curves show a non-monotonic behavior with maximum values of the breakdown voltage for a guard ring pitch between 6 and 7 μm. According to these results, a promising configuration for the termination structures was chosen combining 20 guard rings with a pitch of 6 μm.

TCAD simulations were also performed to estimate the voltage range that needs to be applied to the backside contact for the correct operation of the sensors. As reported in [16], this operating voltage should be larger than the one associated to full depletion, but small enough to avoid an excessive punch through current flow.

**Figure 4:**
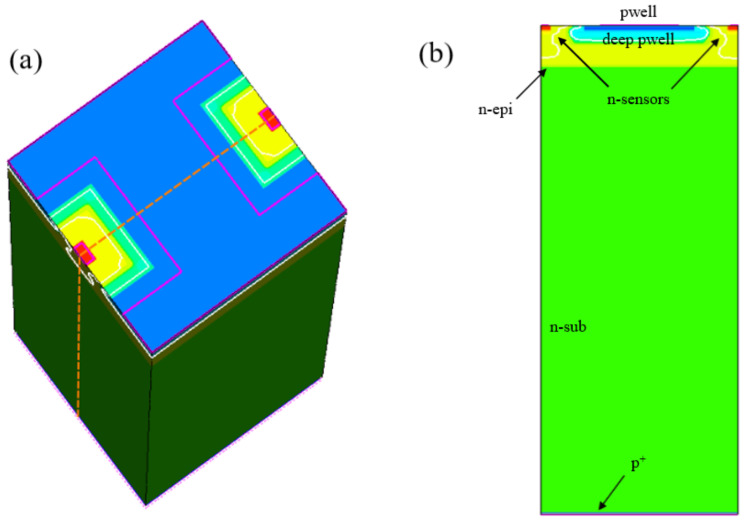
(**a**) 3D domain composed by two pixel halves. The red and blue regions represent the pixel nwells and pwell, respectively. The boundary of the depletion region is marked with white lines. The dashed orange lines represent the edges of the cross section cut plane. (**b**) 2D cross-section (Not to scale).

In the simulations, we exploited the pixel matrix symmetry to reduce the simulation domain size and thus the overall time and resources required for the computation. Figure 4a shows an example 3D simulation domain composed of two pixel halves with a pixel pitch of 25 μm and a substrate thickness of 100 μm, together with a vertical cut plane at the center of the domain. The 2D cross-section obtained in correspondence to the cut plane is shown in Figure 4b. The white lines on the domain represent the boundary of the depletion region for an applied backside voltage equal to the full depletion voltage of −44 V. The yellow region represents the fraction of the surface region free from both nwell and pwell implantations. The surface doping concentration in this region is thus the one of the n-type epitaxial layer, included in the device structure to shift the punch-through onset to higher reverse voltages and thus enable the pixels to operate in full depletion.

The operation voltage range was extracted from I–V curves obtained with quasi-stationary DC simulations. A negative voltage sweep was applied to the backside $p^{+}$ electrode to reverse-bias the substrate. The frontside pwell electrode was connected to the ground reference at 0 V, while the pixel nwells were biased with a positive voltage. A small voltage difference equal to 10 mV was applied between the two nwell sensor electrodes to induce a current flow between them. In fact, while biasing the backside up to the full depletion voltage, a resistive path between the frontside nwells is present, and thus a current can flow between these electrodes as a result of the applied voltage difference. A hole current flows instead between the backside $p^{+}$ electrode and the frontside p-type implants and starts growing exponentially once the backside bias exceeds the onset of punch-through.

**Figure 5:**
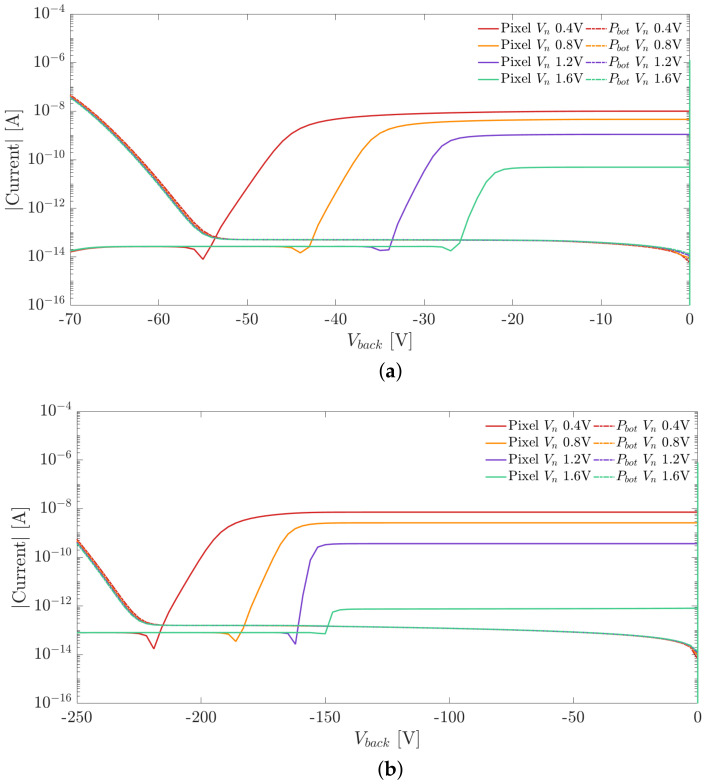
(**a**) Frontside nwell and backside $p^{+}$ implant I–V curves for different values of applied $V_{n}$ in a simulation domain with 25 μm pixel pitch and substrate thickness of 100 μm. (**b**) Frontside nwell and backside $p^{+}$ implant I–V curves for different values of applied $V_{n}$ in a simulation domain with 25 μm pixel pitch and substrate thickness of 300 μm.

Figure 5a,b show the I–V curves of a pixel nwell and of the backside $p^{+}$ implant for different voltages applied to the frontside nwell $V_{n}$ and for two different substrate thicknesses 100 and 300 μm. The dip in the curve, corresponding to the change of sign in the pixel current, was chosen as reference for the extraction of the full depletion voltage. Since the presence of a small punch-through current density can be tolerated and does not compromise the operation of the detector, the upper limit for the bias voltage range was chosen as the point where the backside current exceeds a certain threshold value of $0.6\times 10^{-12}$ A/μm^2^, chosen accordingly to the acceptable power dissipation. It can be observed that the operating range can be extended by increasing the voltage applied to the sensor nodes, and an applied voltage of 0.8 V is sufficient to provide a depletion voltage significantly smaller than the onset of punch-through currents. On the contrary, the current measured at the backside $p^{+}$ implant electrode shows no dependency on the applied $V_{n}$.

## 3. Experimental Measurements

As mentioned in Section 1, the experimental measurements were performed on a batch of samples produced within the first fabrication run. Several test structures were measured in order to fully characterize the samples, which came from different silicon wafers with substrate thicknesses of 100 and 300 μm.

**Figure 6:**
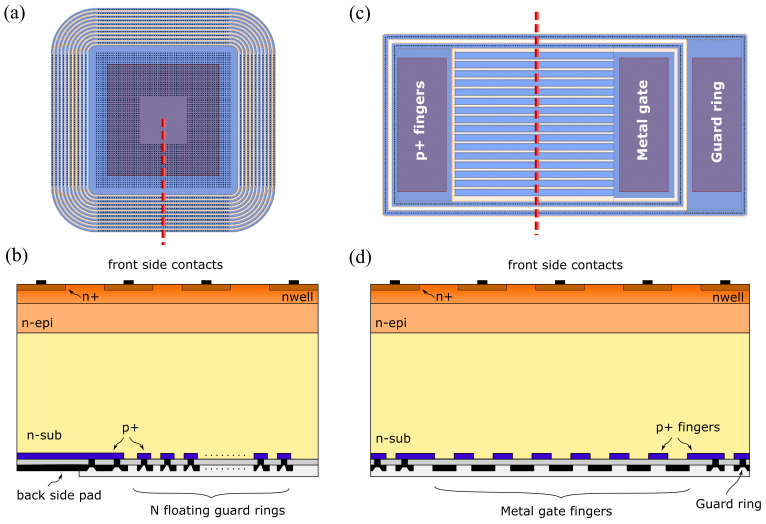
(**a**) Layout of a backside diode with floating guard rings. (**b**) Schematic cross section of a backside diode with floating guard rings. (**c**) Layout of a gated diode with dedicated guard ring. (**d**) Schematic cross section of a gated diode with dedicated guard ring.

The test structures include pixel arrays with integrated electronics and 50 μm pixel pitch and passive test structures made of pixel arrays with 50 μm and 25 μm pitch, having all the sensor nodes connected in parallel to a common metal pad. In addition to active and passive pixel arrays, several test structures have been designed to qualify the characteristics of the backside layers and of the termination structures. More specifically, a set of backside $p^{+}$/n-substrate diodes with different number of guard rings and pitches, designed according to the results of TCAD simulations, were included as control structures in the first fabrication run, together with a MOS capacitor and a gated diode. Figure 6a,c show the simplified layouts of a backside diode with 10 floating guard rings and of a gated diode, while Figure 6b,d represent the schematic cross sections of the same structures shown in correspondence to the cut planes marked with the dashed red lines.

Most of the measurements were performed at a probe station on diced chips using a Keithley 4210 Parameter Analyzer. High-voltage measurements were carried out using a Keithley 2410 Source Meter.

### 3.1. Test Diodes with Floating Guard Rings

The I–V curves of a group of test diodes were acquired to extract the breakdown voltages. These values were compared with the voltages obtained from the TCAD simulations (as described in Section 2) in order to experimentally validate the simulation results. Although the maximum reverse voltage used in the measurements was 800 V, a few of the diodes with 30 guard rings had a breakdown voltage exceeding this value.

**Figure 7:**
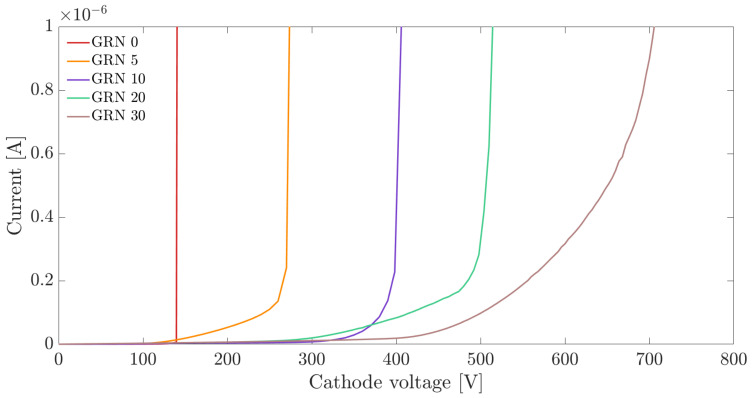
Test diode currents as a function of the applied cathode voltage measured on diodes with different number of guard rings (GRN) and 6 μm pitch.

The I–V curves measured for five diodes with increasing number of guard rings are shown in Figure 7. In the test diodes located near the chip edges, the reverse current includes also the contributions from the generation at the diced chip lateral surface.

The breakdown voltage was estimated as the bias voltage at which the current flowing in the diode exceeds the arbitrarily set threshold current of 1 μA. As it can be clearly noticed, the abrupt increase in the diode current usually associated to the breakdown was not always present; therefore, fixing a threshold on the current is a conservative method for extracting the breakdown voltage. The obtained breakdown voltages measured on a large set of samples are discussed in Section 4.

### 3.2. Gated Diodes

We performed a set of measurements on gate-controlled diodes in order to extract the value of the surface generation velocity at the interface between silicon and the backside passivation layer, which is related to the surface component of the diode leakage current. The measurements were carried out following the procedure explained in [22,23]. A negative constant voltage equal to 5 V was applied to the diode cathode to reverse-bias the region near the backside surface. A voltage sweep was applied to the diode gate to bring the devices from accumulation to strong inversion and the other way around, and the diode current $I_{d}$ was acquired as a function of the applied gate voltage $V_{g}$.

**Figure 8:**
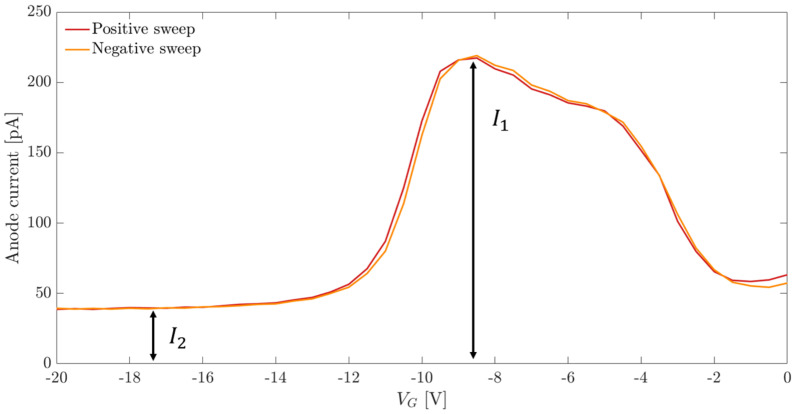
Gated diode anode current as a function of the applied gate voltage.

In Figure 8, where $I_{d}$ is plotted as a function of $V_{g}$, the current can be extracted in different gate operation regions. When the device is in accumulation, the main contribution to the diode current is represented by the leakage current of the diode metallurgical junction [22,23]. Moving to the depletion condition, two additional current contributions due to surface and bulk generation add to the diode current. Finally, once the strong inversion is reached, the surface generation component becomes negligible compared to the metallurgical junction leakage and bulk generation currents.

According to [22,23], the current due to the surface generation $I_{s}$ can be calculated by subtracting the diode current measured in strong inversion condition $I_{2}$ from the maximum diode current in depletion $I_{1}$. The value of the surface generation velocity $s_{0}$ can be estimated from the obtained surface generation current $I_{s}$:

(1) $$s_{0}=\frac{I_{s}}{qn_{i}A_{g}},$$

where the gate area $A_{g}$ was computed as the sum of the finger areas of the comb-like gate structure and *q* and $n_{i}$ represent the elementary charge and the intrinsic concentration of charges at room temperature, respectively.

### 3.3. MOS Capacitors

**Figure 9:**
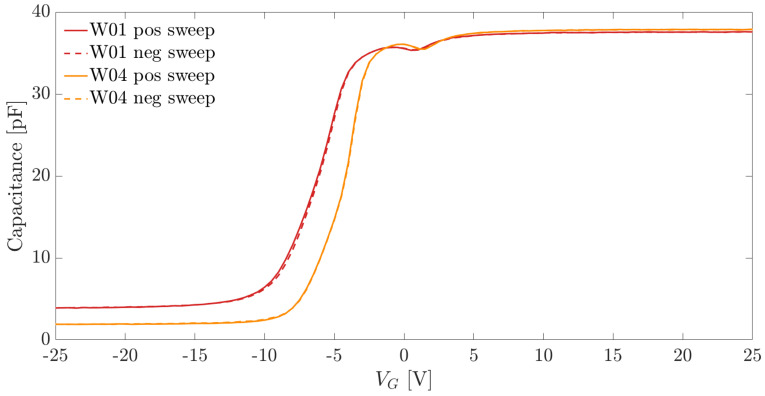
C–V curves of MOS capacitors from two different wafers.

The C–V curves of the MOS capacitors were measured using an AC probe signal with 10 kHz frequency and 100 mV amplitude. The C–V curves obtained from two samples belonging to different wafers are shown in Figure 9, where the continuous and dashed lines represent the C–V curves, which were measured sweeping the bias voltage from strong inversion to accumulation and the other way around.

The thickness of the passivation layer ($t_{ox}$) was extracted from the MOS capacitance in accumulation $C_{ox}$ according to

(2) $$t_{ox}=\frac{C_{ox}}{A_{g}\epsilon_{ox}\epsilon_{0}},$$

where $A_{g}$ is the area of the capacitor, $\epsilon_{0}$ is the permittivity in vacuum, and $\epsilon_{ox}$ is the relative permittivity of the insulator layer.

The concentration of charges in the passivation layer $Q_{ox}$ was also estimated from the C–V curves via

(3) $$Q_{ox}=\frac{-\left(V_{fb}-\phi_{MS}\right)C_{ox}}{qA_{g}};$$

where $V_{fb}$ is the flat band voltage, $\phi_{MS}$ is the metal-to-semiconductor workfunction between aluminum and n-type silicon and $A_{g}$ is the MOS gate area.

### 3.4. Passive Pixel Arrays

A 16 × 18 passive array of pixels with 25 μm pitch, with all the in-pixel sensor nodes connected in parallel, was considered to experimentally extract the operating voltage of the detectors. In this structure, the pixel array is surrounded by an n-type guard ring, which can be biased through a dedicated electrode. Three different electrodes are thus present at the front side: the sensor array, the pwell, which is connected to ground, and the guard ring.

In order to measure the I–V curves of the pixel array, the samples were glued to a support board using a silver-loaded conductive resin. Since the same chip also hosted other backside test structures, in order to avoid contacting the pads of these other structures with the board pad, a thin polymeric film was cut with a computer numerically controlled laser machine and interposed between the chip and and the support board. An opening window with the same dimensions and shape of the pixel array was realized in the polymeric film in order to enable the contact between the board pad and the backside contact of the array.

The needle connectors of the probe station were used to contact the electrodes of the pixel array under test. A negative voltage sweep was applied to the backside electrode, while the voltage applied to the pwell electrode was taken as ground reference. Different voltage levels ($V_{n}$) were applied to the pixel sensor nodes and to the n-guard ring to investigate the influence of $V_{n}$ on the measured I–V curves.

**Figure 10:**
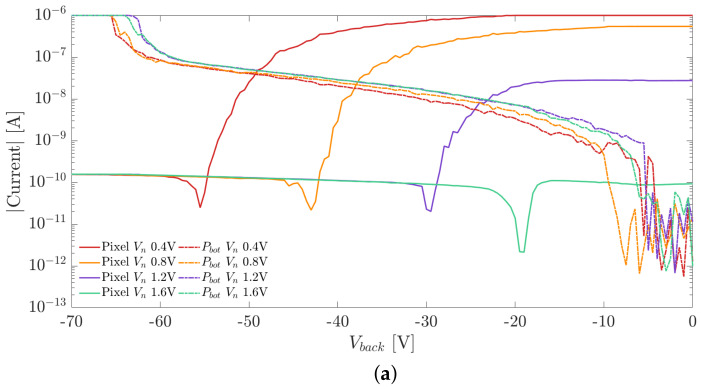

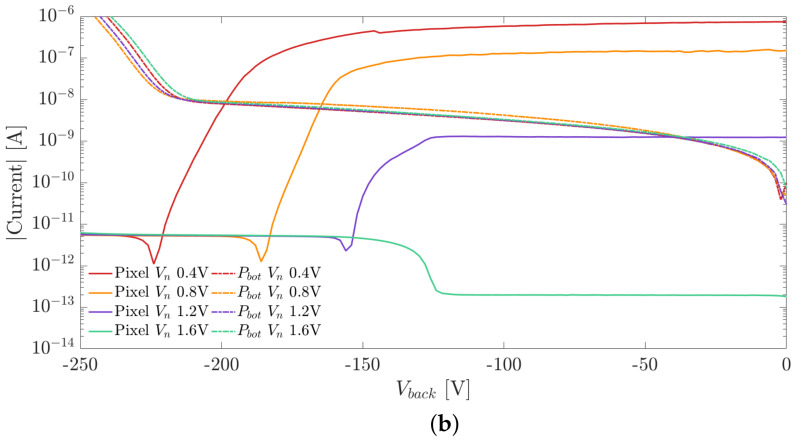
(**a**) Pixel nwell and backside $p^{+}$ implant I–V curves as a function of $V_{n}$ for a substrate thickness of 100 μm. (**b**) Pixel nwell and backside $p^{+}$ implant I–V curves as a function of $V_{n}$ for a substrate thickness of 300 μm.

Figure 10a,b show the I–V curves measured at the pixel nwell and at the backside p-electrode for two samples with different substrate thicknesses. We employed the method explained in Section 2 for the extraction of the full depletion voltage. The curve plateau, which follows the dip marking the onset of the full depletion, represents the pixel leakage current.

The I–V curves measured at the backside $p^{+}$ electrode suffered from the same problem encountered in the measurements of the test diodes. The collected current $I_{Pbot}$ also included the contributions due to the surface generation from the diced chip edge, and the current measured at the backside is dominated by this effect. The onset of punch through was thus estimated as the voltage where the current $I_{Pbot}$ exceeds the threshold of 0.1 μA.

## 4. Experimental Data Analysis and Simulation Results

### 4.1. Backside Surface and Oxide Characteristics

**Figure 11:**
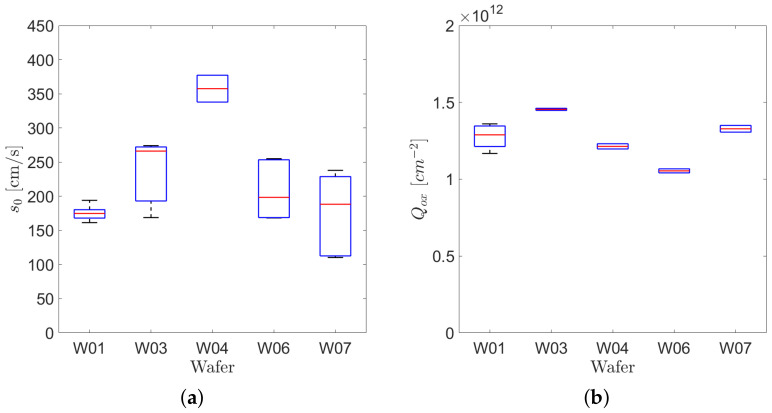
(**a**) Box plot of the surface generation velocities measured on different wafers. (**b**) Box plot of the concentration of charges in the surface passivation layer measured on different wafers.

The surface generation velocities extracted from the gated diodes I–V curves are plotted in Figure 11a for different silicon wafers. The extracted surface generation velocities are in the order of hundreds of cm/s. These values are significantly larger than the ones obtained with thermally grown oxide, and comparable with the ones usually measured on irradiated silicon devices [24].

As explained in Section 3, we extracted the equivalent concentration of oxide charges from the measured C–V characteristics of the MOS capacitors. Figure 11b shows the box plot of the $Q_{ox}$ values obtained on test devices from different wafers. The concentration of positive charges in the insulator layer were between $1\times 10^{12}$ cm^−2^ and $1.5\times 10^{12}$ cm^−2^.

### 4.2. Breakdown Voltages

**Figure 12:**
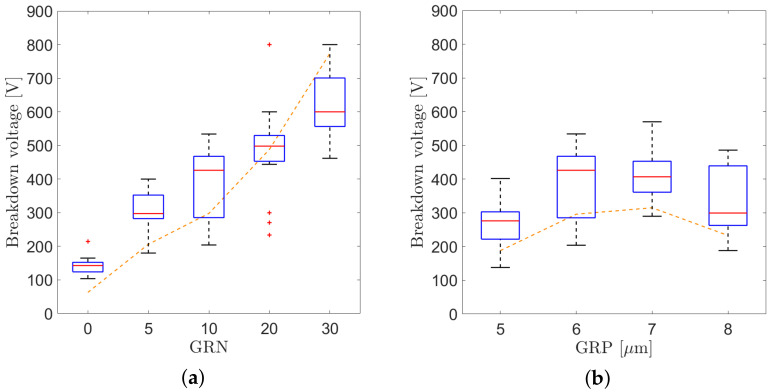
(**a**) Box plot of the breakdown voltages measured on a set of backside diodes from different wafers and breakdown voltages extracted from the simulations (dashed orange line) as a function of the guard ring number for a pitch of 6 μm. (**b**) Box plot of the breakdown voltages measured on backside diodes with 10 guard rings and breakdown voltages extracted from the simulations (dashed orange line) as a function of the guard ring pitch.

The breakdown voltages extracted from the I–V curves measured on the test diodes are reported in two different box plots (Figure 12a,b), where the x-axes represent the number of guard rings and the guard ring pitch, respectively. The maximum and minimum values, the median, the interquartile range, and the outliers are shown in the box plots. The results extracted from a total of 22 devices from seven wafers are plotted on the same graph, and the breakdown voltages obtained from the simulations with *N_ox_* equal to 1 × 10^12^ cm^−2^ are reported for comparison with the dashed orange lines. We noticed no significant differences in the breakdown voltages extracted from devices on different wafers.

As expected, increasing the number of guard rings is an effective way to increase the maximum bias voltage applied at the backside by several hundred volts. The measured results are in good agreement with the ones predicted by simulations and reported in Figure 12 with the dashed orange lines, if an oxide charge of 1 × $10^{12}$ cm^−2^ is considered.

The optimal guard ring pitch was found to be between 6 and 7 μm, which is also in line with the expected behavior observed in the simulations.

### 4.3. Full Depletion and Punch-Through Voltages

**Figure 13:**
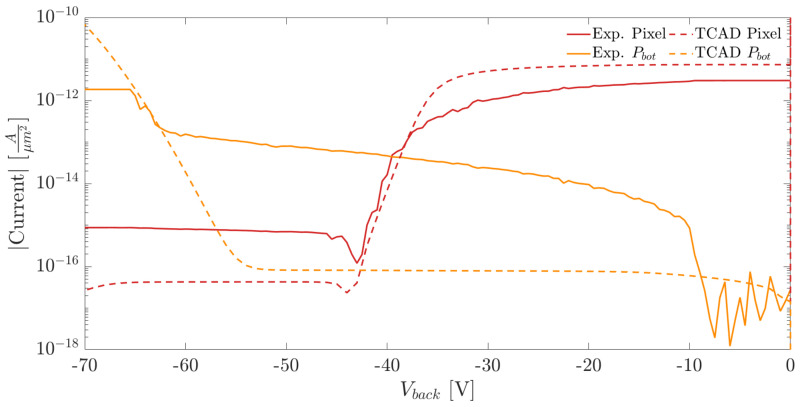
Comparison of the current densities obtained from measurements (Exp.) and simulations (TCAD) for a substrate thickness of 100 μm.

**Figure 14:**
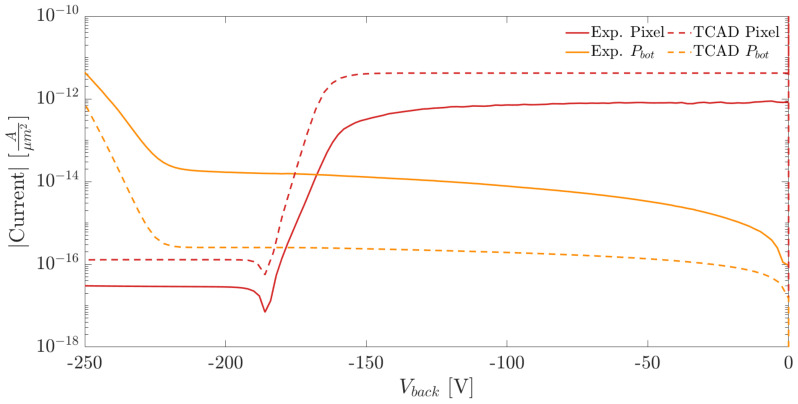
Comparison of the current densities obtained from measurements and simulations for a substrate thickness of 300 μm.

The comparison of the I–V curves measured on the pixel matrix and the ones obtained from the simulations are shown in Figure 13 and Figure 14. The currents were normalized w.r.t. the area in order to provide a meaningful comparison. In the plots we report the results obtained for two substrate thicknesses equal to 100 and 300 μm.

Comparing the I–V curves measured at the pixel nodes (red lines) in Figure 13 and Figure 14, it can be observed that the measured and simulated values of the full depletion voltage are in excellent agreement. On the contrary, in both plots, the intensity of the current flowing at the backside electrode is significantly larger than the simulated one. As mentioned in Section 3, the higher currents obtained experimentally are due to thermal generation at the diced lateral chip surface.

**Figure 15:**
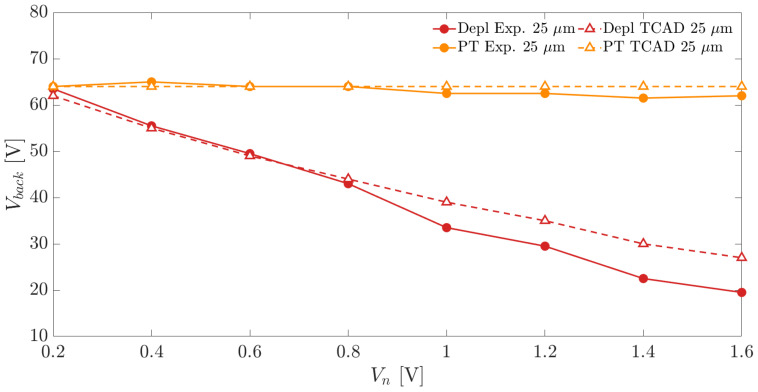
Depletion and punch-through voltages as a function of the applied frontside bias $V_{n}$ for a substrate thickness of 100 μm.

**Figure 16:**
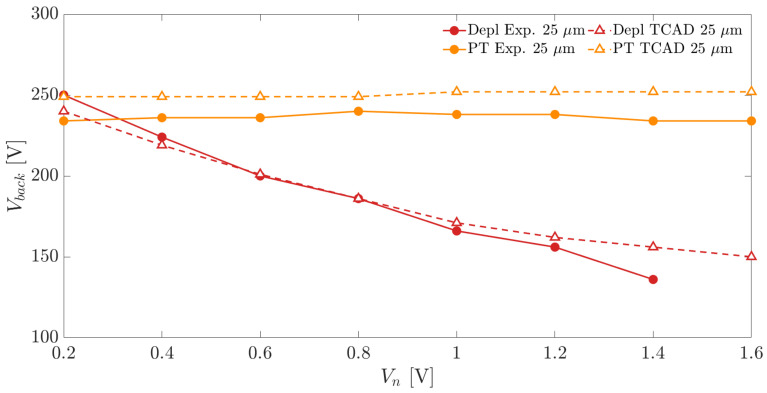
Depletion and punch-through voltages as a function of the applied frontside bias $V_{n}$ for a substrate thickness of 300 μm.

Figure 15 and Figure 16 show the depletion and punch-through voltages, experimental and simulated, for the two substrate thicknesses as a function of the applied $V_{n}$. The results show in both cases a negligible effect of the applied frontside voltage $V_{n}$ on the punch-through onset. On the contrary, the full depletion voltages decrease significantly by increasing the applied $V_{n}$. This determines an increase in the operating voltage range that can be exploited to bias the sensors by simply increasing $V_{n}$. Since, for a safe sensor operation, a sufficient margin between depletion and punch-through voltage needs to be available, the sensors can operate properly when the applied frontside nwell bias is higher than 0.4 V.

It is worth noticing that, thanks to the multiple-guard-ring termination structure surrounding the backside $p^{+}$ pad diode, a high bias voltage, exceeding 200 V, can be applied to the thicker sensors, effectively protecting the backside junction from early breakdown.

## 5. Conclusions

In this work, floating guard ring termination structures for the backside junction needed to bias FD-MAPS detectors have been presented. The structures were designed with the help of TCAD simulations, fabricated with post-processing on front-side processed CMOS wafers and verified experimentally, demonstrating the feasibility of the proposed approach.

A good agreement was found between the breakdown voltages obtained from the simulations and the ones extracted from the I–V curves of the test diodes.

The measurements of the gated diodes and of the MOS capacitors provided an estimation of the surface generation velocity and of the surface oxide charge concentration that can be expected in the future productions realized with this technology.

We are confident that a good radiation hardness can be achieved by the backside diodes realized with this technology, since the test structures proved to be properly working with levels of $s_{0}$ and $N_{ox}$ comparable with the values usually extracted from irradiated devices.

Finally, the operating voltage range of integrated pixels was analyzed both in simulations and experimentally. We investigated the effect of the voltages applied to the frontside nwells and n-guard ring on both full depletion and punch-through voltages. Both the simulations and the measurements showed that increasing the applied $V_{n}$ results in an increased operating range related to the decrease of the full depletion voltage. The inclusion of floating guard rings as termination structures at the backside junction enabled the reliable reverse biasing of devices with thick substrates. In this work, we have experimentally demonstrated the reliable operation of 300 μm pixel sensors. In principle, the termination structures with 30 floating guard rings can grant the application of voltages as high as 400 V, thus extending the reliable operation towards thicker substrates. This option would make this process appealing not only for charged particle and low-energy photon detection, but also for the fabrication of efficient monolithic X-ray pixel detectors with high spatial resolution.

## Data Availability

Experimental data available on request.
